# New estimators for estimating population total: an application to water demand in Thailand under unequal probability sampling without replacement for missing data

**DOI:** 10.7717/peerj.14551

**Published:** 2022-12-13

**Authors:** Chugiat Ponkaew, Nuanpan Lawson

**Affiliations:** 1 Department of Mathematics, Faculty of Science and Technology, Phetchabun Rajabhat University, Phetchabun, Thailand; 2 Department of Applied Statistics, Faculty of Applied Science, King Mongkut’s University of Technology North Bangkok, Bangsue, Bangkok, Thailand

**Keywords:** Water demand, Population total, Unequal probability sampling without replacement, Taylor linearization approach, Nonlinear estimator, Logistic regression, 62D05, 62D10

## Abstract

Water shortage could play an imperative role in the future due to an influx of water demand when compared to water supplies. Inadequate water could damage human life and other aspects related to living. This serious issue can be prevented by estimating the demand for water to bridge the small gap between demand and supplies for water. Some water consumption data recorded daily may be missing and could affect the estimated value of water demand. In this article, new ratio estimators for estimating population total are proposed under unequal probability sampling without replacement when data are missing. Two situations are considered: known or unknown mean of an auxiliary variable and missing data are missing at random for both study and auxiliary variables. The variance and associated estimators of the proposed estimators are investigated under a reverse framework. The proposed estimators are applied to data from simulation studies and empirical data on water demand in Thailand which contain some missing values, to assess the efficacies of the estimators.

## Introduction

Increasing demand for water is highly concerning because of water supply reduction. There are many reasons that cause an increase in water demand such as the rapid growth of the human population, climate change, and so on. The world water resources consist of more water from the sea compared to available fresh water or rainwater. The amount of clean water is also affected by polluted water. Many developing countries face water scarcity and flooding issues due to climate change which can affect their sustainability in economics and lead to unsafe conditions and poor health of the population. Freshwater is used for a myriad of reasons such as household usage, business and industry, agriculture and much more. Thailand is one of the developing countries that mainly uses freshwater in agriculture which accounts for a majority of the usage of the world’s available freshwater. Metropolitan waterworks and provincial waterworks are organizations who are responsible for producing, delivering, and distributing water supply to all provinces in Thailand while also providing resources for water. The former is responsible for Bangkok, Nonthaburi, and Samut Prakan and the latter is responsible for the rest of the country. Some of the consumption of water data are missing in the database system which could lead to the wrong interpretation based on missing data. The missing data or nonresponse should be taken into consideration before processing for further analysis to make for a more powerful interpretation.

If this issue is not addressed, water shortage could lead to repercussions in the future and it would be harmful for human life because of a lack of clean water to use. The management of water resources to avoid facing water scarcity needs to be taken into consideration. Knowledge of the gap between the demand and supply of water could accommodate the strategies and policy planning for the world to be prepared for sustainable water management in order to provide sufficient water according to the demand. Estimating the water demand can benefit future planning to avoid water shortage. Bakker et al. (2014) investigated three models to forecast water demand in both cases with the model using weather input and not using it. The simulation results found that the model using weather input gave a maximum 11 percent of the errors which is essential in water supply system control and detecting irregularity. Huang & Lin (2017) proposed a system dynamics model for studying demand and supply for water resources to avoid water shortage in China. The model had been used to estimate demand and supply for Shandong in China for the next 15 years. Rainwater is also one of the sources of water usage. Boretti & Rosa (2019) examined the correlation between water demand, population growth, and economic growth to estimate water scarcity in the world by 2050. They found that the demand for water is growing even more than the growth of the population and economy along with a low quality of resources and water to use. Kaewprasert, Niwitpong & Niwitpong (2022) proposed confidence intervals for estimation of the mean of delta-gamma distribution using the Bayesian method and applied it to rainfall data in Chiang Mai Thailand.

The biased estimator, namely the ratio estimator is a popular method for estimating population total ( $Y$) or population mean ( $\bar Y$) of a study variable ( $y$) when the information of an auxiliary variable ( $x$) exists and is highly positively correlated with $y$. The ratio estimator was introduced by Cochran (1940) under simple random sampling without replacement (SRSWOR). The mean square error and bias of the ratio estimator are investigated by using the first order approximation of the Taylor linearization approach to transform the ratio estimator to a linear estimator. Then, the properties of the ratio estimator can be approximated from the linear estimator. Sisodia & Dwivedi (1981) proposed a ratio estimator when the population coefficient of variation $({C_x})$ of $x$ is known. The ratio estimator when the kurtosis $({\beta _2}(x))$ is known was proposed by H. P. Singh & M. S. Kakran (1993, unpublished data). Upadhyaya & Singh (1999) suggested the ratio type estimators for estimating population mean when the ${C_x}$ and ${\beta _2}(x)$ are known. Bhushan & Kumar (2022) suggested some classes of population mean estimators based on the optimum value of the constant to improve the efficiency of the estimators under ranked set sampling. The ratio estimators of Cochran (1940), Sisodia & Dwivedi (1981), H. P. Singh & M. S. Kakran (1993, unpublished data) and Upadhyaya & Singh (1999) require the population mean of $x$, $\bar X$ in order to estimate $\bar Y$. Therefore, Perri (2004) proposed an alternative ratio estimator namely regression-in-ratio estimator for estimating $\bar Y$. The estimator of Perri (2004) does not require $\bar X$ by using the regression estimator to estimate this value. In other words, if the auxiliary variable $x$ is correlated with another auxiliary variable namely $u$ then $\bar X$ can be estimated from $u$ by using a regression estimator to estimate this value. The Perri (2004) estimator is a function of two estimators consisting of estimators of $\bar Y$ and $\bar X$. In the context of unequal probability sampling without replacement (UPWOR), Bacanli & Kadilar (2008) modified the ratio estimators under SRSWOR by estimating the population mean of $y$ and $x$ under SRSWOR using the Horvitz & Thompson (1952) type estimators. The variance and associated estimators of Bacanli & Kadilar’s (2008) estimator can be obtained by using a Taylor linearization approach and method from Horvitz & Thompson (1952). Lawson (2021) suggested a general class of ratio estimators for population mean in the form of a combined estimator making use of known auxiliary variables such as the coefficient of variation, coefficient of skewness, coefficient of kurtosis and so on. The Lawson (2021) estimator performed well giving a smaller mean square error especially for a small sample size.

The ratio estimators in the full response case cannot be used to estimate population mean or population total of $y$ when some elements in the sample units are unresponsive. Cochran (1977) considered the ratio estimator under SRSWOR to estimate $\bar Y$ in which information on $x$ is available for all sample units and $\bar X$ is known but some elements of $y$ in the sample units are missing.

Later, ratio estimators with their properties when nonresponse occurs in both $y$ and $x$ but $\bar X$ is known under SRSWOR were proposed by Rao (1986, 1987), Khare & Srivastava (1997), Okafor & Lee (2000), Särndal & Lundström (2005). Kumar (2015), Lawson (2017) introduced estimators for estimating population total and population mean and their variance estimators under probability proportional to size with replacement sampling and nonresponse present in the study. The Lawson estimators are approximately unbiased estimators and they do not require the response propensity when the response probability is uniformly nonresponse, and the sampling fraction is small. Under UPWOR and when information on $x$ is available for all sample units when $\bar X$ is known, Ponkaew & Lawson (2018) proposed a ratio estimator for population total of $y$ with a uniform nonresponse. The variance and associated estimators are also discussed under a reverse framework and when the sampling fraction is ignored. In the same year, Ponkaew (2018) proposed a linear generalized regression estimator (GREG) for population total when information about calibration variables ${u_1},{u_2},...,{u_q}$ exists. The estimator of Ponkaew (2018) is in a form of a nonlinear estimator then automated linearization approach was used to transform this estimator to a linear form. Consequently, the variance and their estimators can be approximated from linear estimators. The ratio estimators in the presence of nonresponse require the value of $\bar X$ in both situations where nonresponse occurs with variables $y$ and $x$ and nonresponse occurs only with the variable $y$. Ponkaew (2018) considered the missing completely at random (MCAR) mechanism which is unlikely to occur in practice. Lawson & Ponkaew (2019) suggested a new GREG estimator using the idea of Lawson (2017) under unequal probability sampling without replacement and nonresponse occurring missing completely at random and when the sampling fraction is small and therefore can be omitted. However, their estimator requires joint inclusion probability which sometimes can be difficult to find. Lawson & Siripanich (2020) improved a new GREG estimator based on the idea of Lawson & Ponkaew (2019) for more flexible situations with the non-uniform nonresponse mechanism or missing at random (MAR) and where the sampling fractions are both large and small. Ponkaew & Lawson (2023) proposed a new approximately unbiased GREG estimator in the form of a ratio estimator following Ponkaew & Lawson (2018) and Lawson & Ponkaew (2019) under the same situation where nonresponse occurs under MCAR but extended it when the sampling fractions are both large and small which is in a general form.

Some researchers suggested to estimate the missing values before further analysis. For example, Shahzad et al. (2020) proposed population mean estimators for when there are some missing observations in the study utilizing robust regression to apply to the regression coefficient estimator under SRSWOR when outliers are present in the study. They considered when nonresponse occurs in the study variable, and in both the study and auxiliary variable when the population mean of the auxiliary variable is known and unknown. Anas et al. (2022) also suggested ratio type regression estimators when nonresponse is present in the study in three situations similar to Shahzad et al. (2020) but the quantile regression in the mean estimator when outliers are present in the study was used. Chodjuntug & Lawson (2022a) suggested a new imputation method to create a population mean estimator when missing data appears in the study variable and applied it to estimate fine particulate matter in Bangkok, Thailand. They suggested to apply two constants to minimize the mean square error of the population mean estimator. Chodjuntug & Lawson (2022b) developed a new estimator by adjusting Chodjuntug & Lawson’s (2022a) by utilizing the response rate and the constant that minimizes the mean square error (MSE) of their proposed estimator. Their estimator using the constant that makes the minimum MSE performed the best. Bhushan et al. (2022) proposed some imputation methods for estimating population mean in the form of logarithmic imputations under SRSWOR for missing data.

In this article, we aim to propose new ratio estimators by extending the Ponkaew & Lawson (2018) estimator to situations where $\bar X$ is known or unknown and nonresponse occurs with both variables $y$ and $x$. In the situation where $\bar X$ is unknown we used the concept from Perri (2004) to estimate its value from the calibration variables ${u_1},{u_2},...,{u_q}$ using the linear GREG estimator of Ponkaew (2018). The variance and associated estimators of the proposed estimators are investigated under the reverse framework. Furthermore, the proposed ratio estimators are considered under both missing at random (MAR) which is more flexible to occur in practice and also considered under MCAR nonresponse mechanism. Finally, we compared the efficiency of the proposed estimators and their variance estimators between the MAR and MCAR mechanisms through a simulation study and an application to water demand data in Thailand.

## Materials and Methods

### Basic setup

In this section, we introduce notations and basic notions about the population total estimator and their variance estimators under the reverse framework. Let $y$ be a study variable and the population total of $y$ is $Y = \sum\limits_{i \in U} {{y_i}}$ where $U = \{ 1,2,...,N\}$ and $N$ is the population size. Suppose the auxiliary variables $x$, $w$ and the size variable $k$ are available and highly positively correlated with the study variable. The calibration variables ${u_1},{u_2},...,{u_q}$ where $q \ge 1$ are also available and they are correlated with the auxiliary variable $x$. Let, ${{ u_ i}} = (\matrix{ 1 & {{u_{i1}}} & { L}& {{u_{iq}}} \cr } {)}^{\prime}$ and ${{\rm U}_N} = (\matrix{ {{{ u_1}}} & {{{ u_2}}} & {L} & {{{ u_N}}} \cr } {)}^{\prime}$ be the $N \times (q + 1)$ matrix values of ${{ u_i}}$. We are using the GREG estimator model from Särndal, Swensson & Wretman (1992) and Särndal (2007) in which the linear assisting model $\xi$, ${E_\xi }({x_i}) = {{ {\beta }}}^{\prime}{{ u}_i}$ and ${V_\xi }({x_i}) = \sigma _i^2$. The linear assisting model $\xi$ is a model describing the relationship between the study variable and auxiliary variable. Let ${q_i}$ be determined by the linear assisting model $\xi$ that is ${q_i} = {1 \mathord{\left/ {\vphantom { {\sigma _i^2}}} \right.} {\sigma _i^2}}$. Usually, the standard choice of ${q_i}$ is ${q_i} = 1$ and it is determined by the linear assisting model $\xi$: ${E_\xi }({x_i}) = { {\beta }^{\prime}}{{ u}_i}$ and ${V_\xi }({x_i}) = {\sigma ^2}$.

Let, ${ F}$ be the set of all possible subsets of $U$ and the sample $s$ of size $n$ was selected from the population $U$ under UPWOR. A sampling design $p(.)$ is a probability distribution on ${ F}$, *i.e*., $P(s) \ge 0$ for all $s \in { F}$ and $\sum\limits_{s \in {\rm F}} {p(s)} = 1$. Let, ${\pi _i} = P(i \in s) = \sum\limits_{s\ni i} {P(s)}$ be the first order inclusion probability and ${\pi _{ij}} = P(i \wedge j \in s) = \sum\limits_{s \supset \{ i,j\} } {P(s)}$ be the second order inclusion probability. We also define ${E_S}( \bullet )$ and ${V_S}( \bullet )$ as the expectation and variance operators with respect to the UPWOR sampling design.

In the presence of nonresponse, let subscript $R$ and ${r_i}$ be the nonresponse mechanism and nonresponse indicator variable of ${y_i}$ which ${r_i} = 1$ if unit $i$ responds to item $y$ otherwise ${r_i} = 0$. Let, ${ R} = (\matrix{ {{r_1}} & {{r_2}} & { L} & {{r_N}} \cr } {)}^{\prime}$ be the vector of the response indicator and ${p_i} = P({r_i} = 1)$ be the response probability under MAR nonresponse. Let, ${E_R}( \bullet )$ and ${V_R}( \bullet )$ be the expectation and variance operators with respect to the nonresponse mechanism. Three assumptions are defined; $({A_1}){\rm }$ the response mechanism is uniform response. $({A_2}){\rm }{{\hat  {\beta } }_r} - {\beta } = {O_p}(n_r^{ - \textstyle{1 \over 2}})$ and $({A_3}){\rm }V\left( {\sum\limits_{i \in s} {\displaystyle{{{b_i}} \over {{\pi _i}}}} } \right) \to 0$ as $n \to \infty$ where ${b_i} = {w_i}$ or ${r_i}$. We also consider three more conditions for investigating the estimator of $Y = \sum\limits_{i \in U} {{y_i}}$ as follows. $({B_1})$ nonresponse occurs only on $y$, the information on ${x_i}$ is available for all $i \in s$ and $\bar X$ is known. $({B_2})$ nonresponse occurs on both $y$ and $x$ and $\bar X$ is known and $({B_3})$ nonresponse occurs both with $y$ and $x$ and $\bar X$ is unknown but information on ${u_1},{u_2},...,{u_q}$ are available for all $i \in s$ and ${\bar U_j} = \displaystyle{1 \over N}\sum\limits_{i \in U} {{u_{ij}}}$, $j = 1,2,...,q$ are known.

Throughout this article, we consider variance estimation of the population total estimator in the presence of nonresponse under the reverse framework. Therefore, we discuss three steps to investigate the variance and its nonlinear estimator such as the ratio estimator when nonresponse occurs in the study variable. Assume that we have $K$ variables consisting of ${t_1}$, the study variable and ${t_2},{t_3},...,{t_K}$, auxiliary variables. Let ${\hat {Y}_s}$ be a nonlinear estimator and be defined by,

(1) $${\hat {Y}_s} = \psi (\hat {\rm T}),$$

where $\psi$ is a known smooth function, $\hat { T} = \left[ {\matrix{ {{{\hat {t}}_1}} & {{{\hat {t}}_2}} & { L}& {{{\hat {t}}_K}} \cr } } \right]$, $K \ge 2$ ${\hat {t}_k} = \sum\limits_{i \in s} {\displaystyle{{{r_i}{t_{ki}}} \over {{\pi _i}{p_i}}}}$ if the variable ${t_k}$ exhibits nonresponse otherwise it can be obtained by ${\hat {Y}_s}.$ Under the reverse framework, variance of ${\hat {t}_k} = \sum\limits_{i \in s} {\displaystyle{{{t_{ki}}} \over {{\pi _i}}}}$

(2) $$V({\hat {Y}_s}) = {E_R}{V_S}\left( {\left. {{{\hat {Y}}_s}} \right|{ R}} \right) + {V_R}{E_S}\left( {\left. {{{\hat {Y}}_s}} \right|{ R}} \right) = {V_1} + {V_2},$$

where ${V_1} = {E_R}{V_S}\left( {\left. {{{\hat {Y}}_s}} \right|{ R}} \right)$ and ${V_2} = {V_R}{E_S}\left( {\left. {{{\hat {Y}}_s}} \right|{ R}} \right)$. The formula of $V({\hat {Y}_s})$ consists of three steps as below.

**Step 1:** Investigate a formula of ${V_1} = {E_R}{V_S}\left( {\left. {{{\hat {Y}}_s}} \right|{ R}} \right)$. Since ${\hat {Y}_s}$ is in a form of a nonlinear estimator then ${V_1} = {E_R}{V_S}\left( {\left. {{{\hat {Y}}_s}} \right|{ R}} \right)$ can be approximated by,

(3) $${{V}^{\prime}_1} \cong {E_R}{V_S}\left( {\left. {{{\hat {Y}}_{s(1)}}} \right|{ R}} \right),$$

where ${\hat {Y}_{s(1)}}$ is a linear estimator of ${\hat {Y}_s}$ under the Taylor linearization approach.

**Step 2:** Investigate the formula of ${V_2} = {V_R}{E_S}\left( {\left. {{{\hat {Y}}_s}} \right|{ R}} \right)$. The formula of ${V_2} = {V_R}{E_S}\left( {\left. {{{\hat {Y}}_s}} \right|{ R}} \right)$ can be approximated by,

(4) $$V_2^\prime  \cong {V_R}\left( {\left. {Y_{_s}^\% } \right|R} \right),$$

where $Y_{_s}^{^\% } = {E_S}\left( {\left. {{{\hat {Y}}_s}} \right|R} \right)$.

**Step 3:** Approximate the value of $V({\hat {Y}_s})$ and its estimator. The value of $V({\hat {Y}_s})$ can be obtained by,

(5) $$V({\hat {Y}_s}) = {{V}^{\prime}_1} + {{V}^{\prime}_2}.$$

The estimator of $V({\hat {Y}_s})$ can be obtained by substituting estimators for the unknown parameter in (5). Then, the estimator of $V({\hat {Y}_s})$ is defined by,

(6) $$\hat {V}({\hat {Y}_s}) = {\hat {V}^{\prime}_1} + {\hat {V}^{\prime}_2},$$

where ${\hat {V}^{\prime}_1}$, ${\hat {V}^{\prime}_2}$ are the estimators of ${{V}^{\prime}_1}$, ${{V}^{\prime}_2}$ respectively.

### Existing estimators under uniform nonresponse

Uniform nonresponse or missing completely at random (MCAR) is a nonresponse mechanism in which the probability of response of the study variable $y$ neither depends on itself nor another variable such as $x,{\rm }k{\rm }$ or $w.$ In this section, we discuss two estimators for estimating population total in the presence of uniform nonresponse namely ratio and GREG estimators proposed by Ponkaew & Lawson (2018) and Ponkaew (2018), respectively. The variance estimation of both ratio and GREG estimators are considered under the reverse framework and the sampling fraction is negligible with the UPWOR sampling design.

### The ratio estimator

When nonresponse occurs only with $y$ but the population mean and its estimator of $x$ are available, Ponkaew & Lawson (2018) proposed ratio estimators to estimate population mean and the total of $y$ under unequal probability sampling without replacement and the nonresponse mechanism is MCAR. The Ponkaew & Lawson (2018) estimator for population mean is

(7) $$\hat {\bar{Y}_R} = \displaystyle{{\displaystyle{1 \over N}\sum\limits_{i \in s} {\displaystyle{{{r_i}{y_i}} \over {{\pi _i}p}}} } \over {\displaystyle{1 \over N}\sum\limits_{i \in s} {\displaystyle{{{x_i}} \over {{\pi _i}}}} }}\bar X = \displaystyle{{{\hat {\bar Y}_r}} \over {{\hat{ \bar X}_{HT}}}}\bar X = {\hat {R}_r}\bar X,$$

where ${\hat{ \bar Y_r}} = \displaystyle{1 \over N}\sum\limits_{i \in s} {\displaystyle{{{r_i}{y_i}} \over {{\pi _i}p}}}$, ${\hat{\bar{X}}_{HT}} = {1 \over N}\sum\limits_{i \in s} {{{x_i} \over{{\pi_{i}}}}} $ and ${\hat {R}_r} = \widehat {{{\bar Y}_r}}{({\hat{\bar{X}}_{HT}})^{ - 1}}$. Ponkaew & Lawson’s (2018) estimator for population total is

(8) $${\hat {Y}_R} = N\hat {\bar Y_R} = N\bar X{\hat {R}_r}.$$

We note that, if $p$ is unknown the estimator of $p$ is equal to $\hat {p} = \left( {\sum\limits_{i \in s} {\displaystyle{{{r_i}} \over {{\pi _i}}}} } \right){\left( {\sum\limits_{i \in s} {\displaystyle{1 \over {{\pi _i}}}} } \right)^{ - 1}}$. The variance and associated estimators of the estimator in (8) is defined in (9),

(9) $$V\left( {{{\hat {Y}}_R}} \right) = \sum\limits_{i \in U} {{D_i}} {({y_i} - R{x_i})^2} + \sum\limits_{i \in U} {\sum\limits_{i\backslash \{ j\} \in U} {{D_{ij}}({y_i} - R{x_i})({y_j} - R{x_j})} } + \sum\limits_{i \in U} {\displaystyle{{1 - {p_i}} \over {{p_i}}}y_i^2},$$

where $R = \bar Y{\bar X^{ - 1}}$. The estimator of $V\left( {{{\hat {Y}}_R}} \right)$ is given in (10),

(10) $$\hat {V}\left( {{{\hat {Y}}_R}} \right) = \sum\limits_{i \in s} {{{\hat {D}}_i}} {({y_i} - {\hat {R}_r}{x_i})^2} + \sum\limits_{i \in s} {\sum\limits_{i\backslash \{ j\} \in s} {{{\hat {D}}_{ij}}({y_i} - {{\hat {R}}_r}{x_i})({y_j} - {{\hat {R}}_r}{x_j})} } + \sum\limits_{i \in s} {{{\hat {E}}_i}y_i^2},$$

where $\hat {R}_r^{} = \sum\limits_{i \in s} {\displaystyle{{{r_i}{y_i}} \over {{\pi _i}p}}} {\left( {\sum\limits_{i \in s} {\displaystyle{{{x_i}} \over {{\pi _i}}}} } \right)^{ - 1}}$, ${\hat {E}_i} = \displaystyle{{{r_i}{E_i}} \over {{\pi _i}}}$, ${\hat {D}_i} = \displaystyle{{{r_i}{D_i}} \over p}$ and ${\hat {D}_{ij}} = \displaystyle{{{r_i}{r_j}{D_{ij}}} \over {{p^2}}}$.

### The GREG estimator

The GREG estimators for estimating population mean or population total of the study variable is a powerful method when the calibration variables ${u_1},{u_2},...,{u_q}$ are present where $q \ge 1$ are also available. In full response, Särndal, Swensson & Wretman (1992) and Särndal (2007) proposed a GREG estimator under the linear assisting model $\xi$,

(11) $${E_{\xi} }({x_i}) = {\beta}^{\prime}{{ u}_i}\,{\rm and}\,{V_{\xi} }({x_{i}}) = \sigma _{i^{2}}.$$

Let ${Q_s} = diag{({q_i})_{s \times s}}$ and ${q_i}$ be determined by the linear assisting model $\xi$ in (5.1) *i.e*., ${q_i} = \sigma _i^{ - 2}$. In the presence of nonresponse, Särndal & Lundström (2005) proposed a linear GREG estimator to estimate population total. They investigated variance and associated estimators under the two-phase framework. Ponkaew (2018) proposed linear GREG estimators for estimating the population mean of $x$ under the MCAR mechanism which is defined by,

(12) $$\eqalign{{\hat{\bar{X}}_{GREG}^{(1)}} = \displaystyle{1 \over N}\sum\limits_{i \in s} {\displaystyle{{{r_i}{x_i}} \over {{\pi _{i}}p}}} + {\left[ {\bar { U} - \displaystyle{1 \over N}\sum\limits_{i \in s} {\displaystyle{{{r_{i}}{{ u}_i}} \over {{\pi _{i}}p}}} } \right]\prime }{\left( {\sum\limits_{i \in s} {\displaystyle{{{r_{i}}{q_{i}}{{ u}_{i}}{{{ {u}^{\prime}}}_{i}}} \over {{\pi _{i}}p}}} } \right)^{ - 1}}\left( {\sum\limits_{i \in s} {\displaystyle{{{r_{i}}{q_{i}}{{ u}_{i}}{x_{i}}} \over {{\pi _{i}}p}}} } \right)\cr= \hat{ \bar{X}_{r}^{(1)} }+ {\left[ {\bar { U} - \hat{\bar{ U}}_{r}^{(1)}} \right]\prime }{\hat { \beta }_{r}},}$$

where ${\hat{\bar{X}}_{r}^{(1)} }= \displaystyle{1 \over N}\sum\limits_{i \in s} {\displaystyle{{{r_{i}}{x_{i}}} \over {{\pi _{i}}p}}} ,$ $\hat{\bar{ U}}_{r}^{(1)} = \displaystyle{1 \over N}\sum\limits_{i \in s} {\displaystyle{{{r_{i}}{{ u}_{i}}} \over {{\pi _{i}}p}}}$, and ${{\hat { \beta }}_{r}} = {\left( {\sum\limits_{i \in s} {\displaystyle{{{r_{i}}{q_{i}}{{ u}_{i}}{{{ {u}^{\prime}}}_{i}}} \over {{\pi _{i}}p}}} } \right)^{ - 1}}\left( {\sum\limits_{i \in s} {\displaystyle{{{r_{i}}{q_{i}}{{ u}_{i}}{x_{i}}} \over {{\pi _{i}}p}}} } \right) = {\left( {\sum\limits_{i \in s} {\displaystyle{{{r_{i}}{q_{i}}{{ u}_{i}}{{{ {u}^{\prime}}}_{i}}} \over {{\pi _{i}}}}} } \right)^{ - 1}}\left( {\sum\limits_{i \in s} {\displaystyle{{{r_{i}}{q_{i}}{{ u}_{i}}{x_{i}}} \over {{\pi _{i}}}}} } \right)$.

Then, the GREG estimator to estimate the population total of $x$ is

(13) $$\eqalign{\hat {X}_{GREG}^{(1)} = N{\hat{\bar{X}}_{GREG}^{(1)}} = \sum\limits_{i \in s} {\displaystyle{{{r_{i}}{x_{i}}} \over {{\pi _{i}}p}}} + {\left[ {{ U} - \sum\limits_{i \in s} {\displaystyle{{{r_{i}}{{ u}_{i}}} \over {{\pi _{i}}p}}} } \right]\prime }{\left( {\sum\limits_{i \in s} {\displaystyle{{{r_{i}}{q_{i}}{{ u}_{i}}{{{ {u}^{\prime}}}_{i}}} \over {{\pi _{i}}p}}} } \right)^{ - 1}}\left( {\sum\limits_{i \in s} {\displaystyle{{{r_{i}}{q_{i}}{{ u}_{i}}{x_{i}}} \over {{\pi _{i}}p}}} } \right),\cr = \hat {X}_{r}^{(1)} + {\left[ {{ U} - {\hat { U}}_{r}^{(1)}} \right]\prime }{{\hat { \beta }}_{r}},}$$

where $\hat {X}_{r}^{(1)} = N{\hat{\bar{X}}_{r}^{(1)}} = \sum\limits_{i \in s} {\displaystyle{{{r_{i}}{x_{i}}} \over {{\pi _{i}}p}}} ,$ ${\hat { U}}_{r}^{(1)} = N\hat{\bar { U}}_{r}^{(1)} = \sum\limits_{i \in s} {\displaystyle{{{r_{i}}{{u}_{i}}} \over {{\pi _{i}}p}}}$.

Under the reverse framework and when sampling fraction is negligible the variance of $\hat {W}_{GREG}^{(1)}$ is

(14) $$V(\hat {X}_{GREG}^{(1)}) \cong \sum\limits_{i \in U} {{D_{1i}}e_{i}^2 + } \sum\limits_{i \in U} {\sum\limits_{j \in U\backslash \{ i\} } {{D_{ij}}{e_{i}}{e_{j}}} },$$

where ${D_{1i}} = \displaystyle{{(1 - {\pi _{i}})} \over {{\pi _{i}}p}}$, ${D_{ij}} = \displaystyle{{{\pi _{ij}} - {\pi _{i}}{\pi _{j}}} \over {{\pi _{i}}{\pi _{j}}}}$ and ${e_{i}} = ({x_{i}} - {{ {u}^{\prime}}_{i}}{ \beta })$.

The estimator of $V(\hat {W}_{GREG}^{(1)})$ is equal to.

(15) $$\hat {V}(\hat {X}_{GREG}^{(1)}) \cong \displaystyle{1 \over {{{{p}^{\prime}}^2}}}\left[ {\sum\limits_{i \in s} {{{\hat {D}}_{i}}{r_{i}}\hat {e}_{i}^2 + } \sum\limits_{i \in s} {\sum\limits_{j \in s\backslash \{ i\} } {{{\hat {D}}_{ij}}{r_{i}}{r_{j}}{{\hat {e}}_{i}}{{\hat {e}}_{j}}} } } \right],$$

where ${\hat {e}_{i}} = ({x_{i}} - {{\rm {u}^{\prime}}_{i}}{{\hat { \beta }}_{r}})$, ${\hat {D}_{i}} = \displaystyle{{1 - {\pi _{i}}} \over {\pi _{i}^2}}$, ${\hat {D}_{ij}} = \displaystyle{{{\pi _{ij}} - {\pi _{i}}{\pi _{j}}} \over {{\pi _{ij}}{\pi _{i}}{\pi _{j}}}}$, ${p}^{\prime} = \left( {\sum\limits_{i \in s} {\displaystyle{1 \over {{\pi _{i}}}}} } \right){\left( {\sum\limits_{i \in s} {\displaystyle{{{r_{i}}} \over {{\pi _{i}}}}} } \right)^{ - 1}}$ if $p$ is unknown otherwise ${p}^{\prime} = p$.

## Results and discussion

### The proposed new ratio estimators

In the previous section, we introduced two estimators of the population total: ratio and GREG estimators in the presence of uniform nonresponse. The variance estimation for both ratio and GREG estimators are considered under the UPWOR sampling design and when the sampling fraction is negligible. However, the ratio estimators in (7) and (8) are considered under a situation where nonresponse occurs in $y$ only and they require the value of the population mean of $x$. Then, in this section we aim to propose new ratio estimators when nonresponse occurs in both variables $y$ and $x$. We also consider two distinct situations of $\bar X$ that are known or unknown. In the situation where $\bar X$ is unknown we estimate it from the calibration variables ${u_1},{u_2},...,{u_{q}}$ using the GREG estimator. In the context of nonresponse, we investigate the proposed ratio estimator under the MAR mechanism because it has weak assumptions and tends to occur in real life more often than the MCAR mechanism. However, we still consider new ratio estimators under the MCAR mechanism for comparing the efficiency of the proposed estimators. First of all, we extended the Ponkaew & Lawson (2018) estimators to the MAR mechanism. The ratio estimator of Ponkaew & Lawson (2018) for estimating population mean under the MAR mechanism is equal to,

(16) $${\hat{\bar{Y}}_{R}^{(1)}} = \displaystyle{{\displaystyle{1 \over N}\sum\limits_{i \in s} {\displaystyle{{{r_{i}}{y_{i}}} \over {{\pi _{i}}{p_{i}}}}} } \over {\displaystyle{1 \over N}\sum\limits_{i \in s} {\displaystyle{{{x_{i}}} \over {{\pi _{i}}}}} }}\bar X = \displaystyle{{\hat{\bar{Y}}_{r}^{(1)}} \over {{\hat {\bar X}_{HT}}}}\bar X = \hat {R}_{r}^{(1)}\bar X,$$

where ${\hat{\bar{Y}}_{r}^{(1)}} = \displaystyle{1 \over N}\sum\limits_{i \in s} {\displaystyle{{{r_{i}}{y_{i}}} \over {{\pi _{i}}{p_{i}}}}}$, ${\hat{\bar{X}}_{HT}} = \displaystyle{1 \over N}\sum\limits_{i \in s} {\displaystyle{{{x_{i}}} \over {{\pi _{i}}}}}$ and $\hat {R}_{r}^{(1)} = {\hat{\bar{Y}}_{r}^{(1)}}{({\hat{\bar{X}}_{HT}})^{ - 1}}$.

Then, the ratio estimator for estimating population total under the MAR mechanism is

(17) $$\hat {Y}_{R}^{(1)} = N{\hat{\bar{Y}}_{R}^{(1)}} = N\bar X\hat {R}_{r}^{(1)},$$

Under the MAR mechanism if ${p_{i}}$ is unknown then it is estimated using the probit or logistic regression models. The variance and associated estimators of $\hat {Y}_{R}^{(1)}$ are discussed in Theorem 4.1.

***Theorem 1*.** *Under condition* $({B_1})$ *with the reverse framework and the nonresponse mechanism is MAR*.

(1) The variance of ${\hat {Y}_{R}^{\prime(1)}}$ is

$V\left( {{\hat {Y}}_{R}^{\prime(1)}} \right) \cong \sum\limits_{i \in U} {{D_{i}}} A_{i}^2 + \sum\limits_{i \in U} {\sum\limits_{i\backslash \{ j\} \in U} {{D_{ij}}{A_{i}}{A_{j}}} } + \sum\limits_{i \in U} {{E_{i}}y_{i}^2},$

where ${A_{i}} = {y_{i}} - R{x_{i}}$, $R = \bar Y{\bar X^{ - 1}}$ and ${E_{i}} = \displaystyle{{1 - {p_{i}}} \over {{p_{i}}}}$.

(2) The estimator of $V\left( {{\hat {Y}}_{R}^{\prime(1)}} \right)$ is

$\hat {V}\left( {{\hat {Y}}_{R}^{\prime(1)}} \right) \cong \sum\limits_{i \in s} {{{\hat {D}}_{i}}} \hat {A}_{i}^{(1)2} + \sum\limits_{i \in s} {\sum\limits_{i\backslash \{ j\} \in s} {{{\hat {D}}_{ij}}\hat {A}_{i}^{(1)}\hat {A}_{j}^{(1)}} } + \sum\limits_{i \in s} {{{\hat {E}}_{i}}y_{i}^2},$

where $\hat {A}_{i}^{(1)} = {y_{i}} - \hat {R}_{r}^{(1)}{x_{i}}$, $\hat {R}_{r}^{(1)} = \sum\limits_{i \in s} {\displaystyle{{{r_{i}}{y_{i}}} \over {{\pi _{i}}{p_{i}}}}} {\left( {\sum\limits_{i \in s} {\displaystyle{{{x_{i}}} \over {{\pi _{i}}}}} } \right)^{ - 1}}$, ${\hat {E}_{i}} = \displaystyle{{{r_{i}}{E_{i}}} \over {{\pi _{i}}}}$, ${\hat {D}_{i}} = \displaystyle{{{r_{i}}{D_{i}}} \over {{p_{i}}}}$ and ${\hat {D}_{ij}} = \displaystyle{{{r_{i}}{r_{j}}{D_{ij}}} \over {{p_{i}}{p_{j}}}}$.

***Proof*.** Let $\hat {Y}_{R}^{(1)}$ be defined in (17). Therefore, variance of $\hat {Y}_{R}^{(1)}$ is

(18) $$V(\hat {Y}_{R}^{(1)}) = V(N\bar X\hat {R}_{r}^{(1)}) = {N^2}{\bar X^2}V(\hat {R}_{r}^{(1)}).$$

Furthermore, the estimator of $V(\hat {Y}_{R}^{(1)})$ can be obtained by,

(19) $$\hat {V}(\hat {Y}_{R}^{(1)}) = {N^2}{\bar X^2}\hat {V}(\hat {R}_{r}^{(1)}).$$

Since $\hat {R}_{r}^{(1)}$ is a nonlinear estimator then the variance of this estimator is equal to,

(20) $$V(\hat {R}_{r}^{(1)}) = {E_{R}}{V_{S}}\left( {\left. {\hat {R}_{r}^{(1)}} \right|{ R}} \right) + {V_{R}}{E_{S}}\left( {\hat {R}_{r}^{(1)}{ R}} \right) = {V_1} + {V_2},$$

where ${V_1} = {E_{R}}{V_{S}}\left( {\left. {\hat {R}_{r}^{(1)}} \right|{ R}} \right)$, ${V_2} = {V_{R}}{E_{S}}\left( {\left. {\hat {R}_{r}^{(1)}} \right|{ R}} \right)$.

**Step 1:** Investigate the formula of ${V_1} = {E_{R}}{V_{S}}\left( {\left. {\hat {R}_{r}^{(1)}} \right|{ R}} \right)$.

By using the Taylor linearization approach the linear estimator of $\hat {R}_{r}^{(1)}$ is

(21) $$\hat {R}_{r}^{(1)}) \equiv {\rm Constant} + \displaystyle{1 \over {N\bar X}}\sum\limits_{i \in s} {\displaystyle{{{{\tilde A}_{i}}} \over {{\pi _{i}}}}},$$

where ${\tilde A_{i}} = \left( {\displaystyle{{{r_{i}}{y_{i}}} \over {{p_{i}}}} - \tilde R_{r}^{(1)}{x_{i}}} \right)$. Then ${V_1} = {E_{R}}{V_{S}}\left( {\left. {\hat {R}_{r}^{(1)})} \right|{ R}} \right)$ can be approximated by,

$\eqalign{{{V}^{\prime}_1} \cong {E_{R}}{V_{S}}\left( {\left. {\hat {R}_{r}^{(1)}} \right|{ R}} \right)= {E_{R}}{V_{S}}\left( {\left. {Constant + \displaystyle{1 \over {N\bar X}}\sum\limits_{i \in s} {\displaystyle{{{{\tilde A}_{i}}} \over {{\pi _{i}}}}} } \right|{ R}} \right)\\= \displaystyle{1 \over {{N^2}{{\bar X}^2}}}{E_{R}}\left( {\left. {\sum\limits_{i \in U} {{D_{i}}} \tilde A_{i}^2 + \sum\limits_{i \in U} {\sum\limits_{i\backslash \{ j\} \in U} {{D_{ij}}{{\tilde A}_{i}}{{\tilde A}_{j}}} } } \right|{ R}} \right)\\= \displaystyle{1 \over {{N^2}{{\bar X}^2}}}\left( {\sum\limits_{i \in U} {{D_{i}}} A_{i}^2 + \sum\limits_{i \in U} {\sum\limits_{i\backslash \{ j\} \in U} {{D_{ij}}{A_{i}}{A_{j}}} } } \right),}$

where ${A_{i}} = {E_{R}}{V_{S}}\left( {\left. {{{\tilde A}_{i}}} \right|{ R}} \right) = {y_{i}} - R{x_{i}}$ and $R = \bar Y{\bar X^{ - 1}}$.

Therefore,

(22) $${{V}^{\prime}_1} \cong \displaystyle{1 \over {{N^2}{{\bar X}^2}}}\left( {\sum\limits_{i \in U} {{D_{i}}} A_{i}^2 + \sum\limits_{i \in U} {\sum\limits_{i\backslash \{ j\} \in U} {{D_{ij}}{A_{i}}{A_{j}}} } } \right).$$

**Step 2:** Investigate the formula of ${V_2} = {V_{R}}{E_{S}}\left( {\left. {\hat {R}_{r}^{(1)}} \right|{ R}} \right)$.

The formula of ${V_2} = {V_{R}}{E_{S}}\left( {\left. {\hat {R}_{r}^{(1)}} \right|{ R}} \right)$ can be approximated by,

$\eqalign{{{V}^{\prime}_2} \cong {V_{R}}{E_{R}}\left( {\left. {\hat {R}_{r}^{(1)}} \right|{ R}} \right) = {V_{R}}{E_{R}}\left( {\left. {\displaystyle{{\displaystyle{1 \over N}\sum\limits_{i \in s} {\displaystyle{{{r_{i}}{y_{i}}} \over {{\pi _{i}}{p_{i}}}}} } \over {\displaystyle{1 \over N}\sum\limits_{i \in s} {\displaystyle{{{x_{i}}} \over {{\pi _{i}}}}} }}} \right|{ R}} \right) = {V_{R}}\left( {\left. {\displaystyle{{\displaystyle{1 \over N}\sum\limits_{i \in U} {\displaystyle{{{r_{i}}{y_{i}}} \over {{p_{i}}}}} } \over {\bar X}}} \right|{ R}} \right)\\= \displaystyle{1 \over {{N^2}{{\bar X}^2}}}\sum\limits_{i \in U} {\displaystyle{{(1 - {p_{i}})y_{i}^2} \over {{p_{i}}}}} = \displaystyle{1 \over {{N^2}{{\bar X}^2}}}\sum\limits_{i \in U} {{E_{i}}y_{i}^2}}$

where ${E_{i}} = \displaystyle{{(1 - {p_{i}})} \over {{p_{i}}}}$.

Then,

(23) $${{V}^{\prime}_2} \cong \displaystyle{1 \over {{N^2}{{\bar X}^2}}}\sum\limits_{i \in U} {{E_{i}}y_{i}^2}.$$

**Step 3:** Approximate the value of $V(\hat {R}_{r}^{(1)})$ and its estimators.

The value of $V(\hat {R}_{r}^{(1)})$ can be approximated by,

(24) $$V(\hat {R}_{r}^{(1)}) \cong {{V}^{\prime}_1} + {{V}^{\prime}_2}\\= \displaystyle{1 \over {{N^2}{{\bar X}^2}}}\left( {\sum\limits_{i \in U} {{D_{i}}} A_{i}^2 + \sum\limits_{i \in U} {\sum\limits_{i\backslash \{ j\} \in U} {{D_{ij}}{A_{i}}{A_{j}} + \sum\limits_{i \in U} {{E_{i}}y_{i}^2} } } } \right).$$

The estimator of $V(\hat {R}_{r}^{(1)})$ is

(25) $$\hat {V}\left( {\hat {R}_{r}^{(1)}} \right) = \displaystyle{1 \over {{N^2}{{\bar X}^2}}}\left( {\sum\limits_{i \in s} {{{\hat {D}}_{i}}} \hat {A}_{i}^{(1)2} + \sum\limits_{i \in s} {\sum\limits_{i\backslash \{ j\} \in s} {{{\hat {D}}_{ij}}\hat {A}_{i}^{(1)}\hat {A}_{j}^{(1)}} } + \sum\limits_{i \in s} {{{\hat {E}}_{i}}y_{i}^2} } \right).$$

Replace (25) into (18) then the variance of $\hat {Y}_{R}^{(1)}$ is

(26) $$V(\hat {Y}_{R}^{(1)}) \cong \sum\limits_{i \in U} {{D_{i}}} A_{i}^2 + \sum\limits_{i \in U} {\sum\limits_{i\backslash \{ j\} \in U} {{D_{ij}}{A_{i}}{A_{j}} + \sum\limits_{i \in U} {{E_{i}}y_{i}^2} } }.$$

Furthermore, the estimator of $V(\hat {Y}_{R}^{(1)})$ can be obtained by substituting (26) in (19) then,

(27) $$\hat {V}(\hat {Y}_{R}^{(1)}) \cong \sum\limits_{i \in s} {{{\hat {D}}_{i}}} \hat {A}_{i}^{(1)2} + \sum\limits_{i \in s} {\sum\limits_{i\backslash \{ j\} \in s} {{{\hat {D}}_{ij}}\hat {A}_{i}^{(1)}\hat {A}_{j}^{(1)}} } + \sum\limits_{i \in s} {{{\hat {E}}_{i}}y_{i}^2}.$$

In (16) and (17), we extend the ratio estimators of Ponkaew & Lawson (2018) to the MAR mechanism and discussed the variance and its estimators in Theorem 1. However, the ratio estimator for population mean in (16) and for population total in (17) can be used under the condition $({B_1})$ that is, when nonresponse occurs only with the $y$ variable but information on ${x_{i}}$ for all $i \in s$ and $\bar X$ needs to be known. Next, we proposed new ratio estimators under condition $({B_2})$ where nonresponse occurs on both $y$ and $x$ but $\bar X$ is known and condition $({B_3})$ nonresponse occurs both $y$ and $x$ and $\bar X$ is unknown but information of ${u_1},{u_2},...,{u_{q}}$ are available for all $i \in s$ and the population mean of ${u_1},{u_2},...,{u_{q}}$ are also known.

### The new ratio estimator when $\bar X$ is known

Assume that the condition $({B_2})$ is satisfied when nonresponse occurs with both variables $y$ and $x$ but $\bar X$ is known. The new ratio estimator for estimating population mean is given below,

(28) $$\hat {\bar Y}_{R}^{(2)} = \left( {\displaystyle{{\displaystyle{1 \over N}\sum\limits_{i \in s} {\displaystyle{{{r_{i}}{y_{i}}} \over {{\pi _{i}}{p_{i}}}}} } \over {\displaystyle{1 \over N}\sum\limits_{i \in s} {\displaystyle{{{r_{i}}{x_{i}}} \over {{\pi _{i}}{p_{i}}}}} }}} \right)\displaystyle{1 \over N}\sum\limits_{i \in U} {{x_{i}}} = \displaystyle{{{\hat{ \bar Y}_{r}}} \over {{\hat{ \bar X}_{r}}}}\bar X = \hat {R}_{r}^{(2)}\bar X,$$

where ${\hat{\bar{Y}}_{r}} = \displaystyle{1 \over N}\sum\limits_{i \in s} {\displaystyle{{{r_{i}}{y_{i}}} \over {{\pi _{i}}{p_{i}}}}}$, $\hat {\bar X_{r}} = \displaystyle{1 \over N}\sum\limits_{i \in s} {\displaystyle{{{r_{i}}{x_{i}}} \over {{\pi _{i}}{p_{i}}}}}$, $\hat {R}_{r}^{(2)} = {\hat{\bar{Y}}_{r}}{({\hat{\bar{X}}_{r}})^{ - 1}}$. Furthermore, the estimator of ${p_{i}}$ can be obtained by using the probit or logistic regression models under the MAR mechanism. Then, the new ratio estimator for the population total is

(29) $${\hat {Y}}_{R}^{\prime(2)} = N{\hat{\bar{Y}}_{R}^{(2)}} = N\bar X\hat {R}_{r}^{(2)}.$$

The variance and associated estimators of ${\hat {Y}}_{R}^{\prime(2)}$ are discussed in Theorem 2.

***Theorem 2*.** *Under condition* $({B_2})$ *with reverse framework and where* the *nonresponse mechanism is MAR*.

(1) The variance of ${\hat {Y}}_{R}^{\prime(2)}$ is

(30) $$V\left( {{\hat {Y}}_{R}^{\prime(2)}} \right) = \sum\limits_{i \in U} {{D_{i}}} A_{i}^2 + \sum\limits_{i \in U} {\sum\limits_{i\backslash \{ j\} \in U} {{D_{ij}}{A_{i}}{A_{j}}} } + \sum\limits_{i \in U} {{E_{i}}y_{i}^2},$$

where ${A_{i}} = {y_{i}} - R{x_{i}}$, $R = \bar Y{\bar X^{ - 1}}$ and ${E_{i}} = \displaystyle{{1 - {p_{i}}} \over {{p_{i}}}}$.

(2) The estimator of $V\left( {{\hat {Y}}_{R}^{\prime(2)}} \right)$ is

(31) $$\hat {V}\left( {{\hat {Y}}_{R}^{\prime(2)}} \right) = \sum\limits_{i \in s} {{{\hat {D}}_{i}}} \hat {A}_{i}^{(2)2} + \sum\limits_{i \in s} {\sum\limits_{i\backslash \{ j\} \in s} {{{\hat {D}}_{ij}}\hat {A}_{i}^{(2)}\hat {A}_{j}^{(2)}} } + \sum\limits_{i \in s} {{{\hat {E}}_{i}}y_{i}^2},$$

where $\hat {A}_{i}^{(2)} = {y_{i}} - \hat {R}_{r}^{(2)}{x_{i}}$, $\hat {R}_{r}^{(2)} = \sum\limits_{i \in s} {\displaystyle{{{r_{i}}{y_{i}}} \over {{\pi _{i}}{{{p}^{\prime}}_{i}}}}} {\left( {\sum\limits_{i \in s} {\displaystyle{{{r_{i}}{x_{i}}} \over {{\pi _{i}}{p}^{\prime}}}} } \right)^{ - 1}}$, ${\hat {D}_{i}} = \displaystyle{{{r_{i}}{D_{i}}} \over {{\pi _{i}}{{{p}^{\prime}}_{i}}}}$, ${\hat {D}_{ij}} = \displaystyle{{{r_{i}}{D_{ij}}} \over {{\pi _{i}}{{{p}^{\prime}}_{i}}}}$, ${\hat {E}_{i}} = \displaystyle{{{r_{i}}{E_{i}}} \over {{\pi _{i}}{{{p}^{\prime}}_{i}}}}$. The value of ${{p}^{\prime}_{i}}$, ${{p}^{\prime}_{i}} = {p_{i}}$ if ${p_{i}}$ is known otherwise ${{p}^{\prime}_{i}} = {\hat {p}_{i}}$. ${\hat {p}_{i}}$ is the estimator of ${p_{i}}$ from the probit or logistic regression models.

The proof in Theorem 2 is similar to the proof in Theorem 1.

In Theorem 2 we investigated the variance and its estimators of ${\hat {Y}}_{R}^{\prime(2)}$. We note that the variance formulas ${\hat {Y}}_{R}^{\prime(1)}$ and ${\hat {Y}}_{R}^{\prime(2)}$ are the same but the variance estimators of ${\hat {Y}}_{R}^{\prime(1)}$ and ${\hat {Y}}_{R}^{\prime(2)}$ are slightly different because the estimators of ${A_{i}} = {y_{i}} - R{x_{i}}$ are different.

In (28) and (29) we proposed new ratio estimators for population mean and population total of the study variable when nonresponse occurs on both $y$ and $x$ variables but $\bar X$ is known under the MAR mechanism. Furthermore, the variance and its estimator are also discussed in Theorem 2. Next, we proposed the special case of ${\hat {Y}}_{R}^{\prime(2)}$ when the response probability is consider under the MCAR mechanism ( ${p_{i}} = p$ for all $i \in U$). Under the MAR mechanism the population mean estimator is equal to

(32) $$\hat{\bar Y}_{R}^{\prime \prime(2)} = \left( {\displaystyle{{\displaystyle{1 \over N}\sum\limits_{i \in s} {\displaystyle{{{r_{i}}{y_{i}}} \over {{\pi _{i}}p}}} } \over {\displaystyle{1 \over N}\sum\limits_{i \in s} {\displaystyle{{{r_{i}}{x_{i}}} \over {{\pi _{i}}p}}} }}} \right)\displaystyle{1 \over N}\sum\limits_{i \in U} {{x_{i}}} = \left( {\displaystyle{{\displaystyle{1 \over N}\sum\limits_{i \in s} {\displaystyle{{{r_{i}}{y_{i}}} \over {{\pi _{i}}}}} } \over {\displaystyle{1 \over N}\sum\limits_{i \in s} {\displaystyle{{{r_{i}}{x_{i}}} \over {{\pi _{i}}}}} }}} \right)\displaystyle{1 \over N}\sum\limits_{i \in U} {{x_{i}}} = \displaystyle{{{\hat{\bar{Y}}_{r}^{\prime}}} \over {{\hat{\bar{X}}_{r}^{\prime}}}}\bar{X} = {\hat {R}}^{\prime(2)}_{r}\bar X,$$

where ${\hat{ \bar Y}^{\prime}_{r}} = \displaystyle{1 \over N}\sum\limits_{i \in s} {\displaystyle{{{r_{i}}{y_{i}}} \over {{\pi _{i}}}}}$, ${\hat{ \bar X}^{\prime}_{r}} = \displaystyle{1 \over N}\sum\limits_{i \in s} {\displaystyle{{{r_{i}}{x_{i}}} \over {{\pi _{i}}}}}$, ${\hat {R}}_{r}^{\prime(2)} = {\hat {\bar Y}_{r}^{\prime}}{({\hat{ \bar X}_{r}^{\prime}})^{ - 1}}$. Then, the population total estimator is

(33) $${{\hat {Y}}}_{R}^{\prime\prime(2)} = N{\hat{ \bar Y}}_{R}^{\prime\prime(2)} = N\bar X{\hat {R}}_{r}^{\prime(2)}.$$

Finally, the variance and associated estimators of y are discussed in Lemma 3.

***Lemma 3*.** *Under condition* $({B_2})$ *with reverse framework and where the nonresponse mechanism is MCAR*.

(1) The variance of ${\hat {Y}}_{R}^{\prime\prime(2)}$ is

(34) $$V\left( {{\hat {Y}}_{R}^{\prime\prime(2)}} \right) = \sum\limits_{i \in U} {{D_{i}}} A_{i}^2 + \sum\limits_{i \in U} {\sum\limits_{i\backslash \{ j\} \in U} {{D_{ij}}{A_{i}}{A_{j}}} } + \sum\limits_{i \in U} {{{{E}^{\prime}}_{i}}y_{i}^2},$$

where ${A_{i}} = {y_{i}} - R{x_{i}}$, $R = \bar Y{\bar X^{ - 1}}$ and ${{E}^{\prime}_{i}} = \displaystyle{{1 - p} \over p}$.

(2) The estimator of $V\left( {{\hat {Y}}_{R}^{\prime\prime(2)}} \right)$ is

(35) $$\hat {V}\left( {{\hat {Y}}_{R}^{\prime\prime(2)}} \right) = \sum\limits_{i \in s} {{{\hat {D}}_{i}}} {\hat {A}}_{i}^{\prime(2)2} + \sum\limits_{i \in s} {\sum\limits_{i\backslash \{ j\} \in s} {{{\hat {D}}_{ij}}{\hat {A}}_{i}^{\prime(2)}{\hat {A}}_{j}^{\prime(2)}} } + \sum\limits_{i \in s} {{{{\hat {E}}}_{i}^{\prime}}y_{i}^2},$$

where ${\hat {A}}_{i}^{\prime(2)} = {y_{i}} - {\hat {R}}_{r}^{\prime(2)}{x_{i}}$, ${\hat {R}}_{r}^{\prime(2)} = \sum\limits_{i \in s} {\displaystyle{{{r_{i}}{y_{i}}} \over {{\pi _{i}}}}} {\left( {\sum\limits_{i \in s} {\displaystyle{{{r_{i}}{x_{i}}} \over {{\pi _{i}}}}} } \right)^{ - 1}}$, ${\hat {D}_{i}} = \displaystyle{{{r_{i}}{D_{i}}} \over {{\pi _{i}}{p}^{\prime}}}$, ${\hat {D}_{ij}} = \displaystyle{{{r_{i}}{D_{ij}}} \over {{\pi _{i}}{p}^{\prime}}}$, ${\hat {E}_{i}} = \displaystyle{{{r_{i}}{E_{i}}} \over {{\pi _{i}}{p}^{\prime}}}$. The value of ${p}^{\prime}$, ${p}^{\prime} = p$ if $p$ is known otherwise $p = \hat {p}$. $\hat {p}$ is the estimator of $p$ under the MCAR mechanism that is $\hat {p} = \left( {\sum\limits_{i \in s} {\displaystyle{{{r_{i}}} \over {{\pi _{i}}}}} } \right){\left( {\sum\limits_{i \in s} {\displaystyle{1 \over {{\pi _{i}}}}} } \right)^{ - 1}}$.

### The new ratio estimator when $\bar X$ is unknown

Assume that the condition $({B_3})$ is satisfied, $\bar X$ is unknown and nonresponse occurs on both $y$ and $x$ variables. However, the information of variable ${u_1},{u_2},...{u_{q}}$ is available for all $i \in s$ and $\bar { U}$ is known. Furthermore, variables ${u_1},{u_2},...{u_{q}}$ are highly correlated with $x$. Then, we extended the GREG estimator of Ponkaew’s (2018) to the MAR mechanism and it is defined by

(36) $${\hat{\bar{X}}_{GREG}} = {\hat{\bar{X}}_{r}} + {\left[ {\bar { U} - \hat{\bar {U}}_{r}} \right]\prime }{\hat {\beta }_{r}},$$

where ${\hat{\bar{X}}_{r}}= \displaystyle{1 \over N}\sum\limits_{i \in s} {\displaystyle{{{r_{i}}{x_{i}}} \over {{\pi _{i}}{p_{i}}}}} ,$ $\hat{\bar { U}}_{r}^{} = \displaystyle{1 \over N}\sum\limits_{i \in s} {\displaystyle{{{r_{i}}{{\rm u}_{i}}} \over {{\pi _{i}}{{{p}{\prime}}_{i}}}}}$, ${\hat{\bar { U}}} = \displaystyle{1 \over N}\sum\limits_{i \in U} {{{ u}_{i}}}$, ${{\hat { \beta }}_{r}} = {\left( {\sum\limits_{i \in s} {\displaystyle{{{r_{i}}{q_{i}}{{ u}_{i}}{{{ {u}^{\prime}}}_{i}}} \over {{\pi _{i}}{p_{i}}}}} } \right)^{ - 1}}\left( {\sum\limits_{i \in s} {\displaystyle{{{r_{i}}{q_{i}}{{ u}_{i}}{w_{i}}} \over {{\pi _{i}}{p_{i}}}}} } \right)$.

The new ratio estimator for population mean is

(37) $${\hat{\bar{Y}}_{R}^{\prime(3)}} = \displaystyle{{\displaystyle{1 \over N}\sum\limits_{i \in s} {\displaystyle{{{r_{i}}{y_{i}}} \over {{\pi _{i}}{p_{i}}}}} } \over {\displaystyle{1 \over N}\sum\limits_{i \in s} {\displaystyle{{{r_{i}}{x_{i}}} \over {{\pi _{i}}{p_{i}}}}} }}{\hat{\bar{X}}_{GREG}}.$$

Then, the new ratio estimator for population total is

(38) $${\hat {Y}}_{R}^{\prime(3)} = N\hat {\bar Y}_{R}^{\prime(3)}.$$

The variance and associated estimators of ${\hat {Y}}_{R}^{\prime(3)}$ are discussed in Theorem 4.

***Theorem 4*.** *Under condition* $({B_3})$ *with reverse framework and nonresponse mechanism is MAR*.

(1) The variance of ${\hat {Y}}_{R}^{\prime(3)}$ is

(39) $$V\left( {{\hat {Y}}_{R}^{\prime(3)}} \right) = \sum\limits_{i \in U} {{D_{i}}} B_{i}^2 + \sum\limits_{i \in U} {\sum\limits_{i\backslash \{ j\} \in U} {{D_{ij}}{B_{i}}{B_{j}}} } + \sum\limits_{i \in U} {{E_{i}}B_{i}^2},$$

where ${D_{i}} = \displaystyle{{1 - {\pi _{i}}} \over {{\pi _{i}}}}$, ${D_{ij}} = \displaystyle{{{\pi _{ij}} - {\pi _{i}}{\pi _{j}}} \over {{\pi _{i}}{\pi _{j}}}}$, ${E_{i}} = \displaystyle{{1 - {p_{i}}} \over {{p_{i}}}}$ and ${B_{i}} = {x_{i}} -  R{{ {u}^{\prime}}_{i}}{ \beta }$.

(2) The estimator of $V\left( {{\hat {Y}}_{R}^{\prime(3)}} \right)$ is

(40) $$\hat {V}\left( {{\hat {Y}}_{R}^{\prime(3)}} \right) = \sum\limits_{i \in s} {{{\hat {D}}_{i}}} \hat {B}_{i}^2 + \sum\limits_{i \in s} {\sum\limits_{i\backslash \{ j\} \in s} {{{\hat {D}}_{ij}}{{\hat {B}}_{i}}{{\hat {B}}_{j}}} } + \sum\limits_{i \in s} {{{\hat {E}}_{i}}\hat {B}_{i}^2},$$

where ${\hat {D}_{i}} = \displaystyle{{{r_{i}}{D_{i}}} \over {{\pi _{i}}{{{p}^{\prime}}_{i}}}}$, ${\hat {D}_{ij}} = \displaystyle{{{r_{i}}{D_{ij}}} \over {{\pi _{i}}{{{p}^{\prime}}_{i}}}}$, ${\hat {E}_{i}} = \displaystyle{{{r_{i}}{E_{i}}} \over {{\pi _{i}}{{{p}^{\prime}}_{i}}}}$ and ${\hat {B}_{i}} = {x_{i}} - {\hat {R}_{r}}{{ {u}^{\prime}}_{i}}{{\hat { \beta }}_{r}}$, ${{p}^{\prime}_{i}} = {p_{i}}$ if ${p_{i}}$ is known otherwise ${{p}^{\prime}_{i}} = {\hat {p}_{i}}$. ${\hat {p}_{i}}$ is the estimator of ${p_{i}}$ from the probit or logistic regression models.

**Proof.** Let ${\hat {Y}}_{R}^{\prime(3)}$ be defined in (38). However, the new ratio estimator ${\hat{\bar{Y}}_{R}^{(3)}}$ is a function of the GREG estimator ${\hat{\bar{X}}_{GREG}}$ then we use the modified automated linearization approach transform ${\hat{\bar{X}}_{GREG}}$ to a simple form and it is defined by

(41) $${\hat{\bar{X}}_{GREG(1)}} = {\bar { U}^{\prime}\beta + }\displaystyle{1 \over N}\sum\limits_{i \in s} {\displaystyle{{{r_{i}}{C_{i}}} \over {{\pi _{i}}{p_{i}}}}},$$

where ${C_{i}} = {x_{i}} - {{ {u}^{\prime}}_{i}}{ \beta }$. Then, the new ratio estimator ${\hat {\bar Y}}_{R}^{\prime(3)}$ can be approximated by,

(42) $$\hat {\bar Y}_{R(1)}^{\prime(3)} \cong \displaystyle{{\displaystyle{1 \over N}\sum\limits_{i \in s} {\displaystyle{{{r_{i}}{y_{i}}} \over {{\pi _{i}}{p_{i}}}}} } \over {\displaystyle{1 \over N}\sum\limits_{i \in s} {\displaystyle{{{r_{i}}{x_{i}}} \over {{\pi _{i}}{p_{i}}}}} }}{\hat{\bar{X}}_{GREG(1)}}.$$

Therefore, variance of ${\hat {Y}}_{R}^{\prime(3)}$ can be approximated from,

(43) $$V({\hat {Y}}_{R}^{\prime(3)}) = V(N\hat {\bar Y}_{R}^{\prime(3)}) = {N^2}V(\hat {\bar Y}_{R}^{\prime(3)}) \cong {N^2}V(\hat{ \bar Y}_{R(1)}^{\prime (3)}).$$

Furthermore, the estimator of $V({\hat {Y}}_{R}^{\prime(3)})$ can be obtained by,

(44) $$\hat {V}({\hat {Y}}_{R}^{\prime(3)}) \cong {N^2}\hat {V}(\hat{ \bar Y}_{R(1)}^{\prime(3)}).$$

We note that $\hat{ \bar Y}_{R(1)}^{\prime(3)}$ is a nonlinear estimator then we use steps (1) to (5) for investigating the value of $V(\hat {\bar Y}_{R(1)}^{\prime(3)})$ and it is defined by,

(45) $$V\left( {{\hat {Y}}_{R(1)}^{\prime(3)}} \right) \cong \displaystyle{1 \over {{N^2}}}\left[ {\sum\limits_{i \in U} {{D_{i}}} B_{i}^2 + \sum\limits_{i \in U} {\sum\limits_{i\backslash \{ j\} \in U} {{D_{ij}}{B_{i}}{B_{j}}} } + \sum\limits_{i \in U} {{E_{i}}B_{i}^2} } \right],$$

where ${B_{i}} = {x_{i}} -  R{{ {u}^{\prime}}_{i}}{ \beta }$. Substitute (45) into (43) then,

(46) $$V({\hat {Y}}_{R}^{\prime(3)}) \cong \sum\limits_{i \in U} {{D_{i}}} B_{i}^2 + \sum\limits_{i \in U} {\sum\limits_{i\backslash \{ j\} \in U} {{D_{ij}}{B_{i}}{B_{j}}} } + \sum\limits_{i \in U} {{E_{i}}B_{i}^2}.$$

Furthermore, the estimator of $V({\hat {Y}}_{R}^{\prime(3)})$ is

(47) $$\hat {V}\left( {{\hat {Y}}_{R}^{\prime(3)}} \right) = \sum\limits_{i \in s} {{{\hat {D}}_{i}}} \hat {B}_{i}^2 + \sum\limits_{i \in s} {\sum\limits_{i\backslash \{ j\} \in s} {{{\hat {D}}_{ij}}{{\hat {B}}_{i}}{{\hat {B}}_{j}}} } + \sum\limits_{i \in s} {{{\hat {E}}_{i}}\hat {B}_{i}^2},$$

where ${\hat {D}_{i}} = \displaystyle{{{r_{i}}{D_{i}}} \over {{\pi _{i}}{{{p}^{\prime}}_{i}}}}$, ${\hat {D}_{ij}} = \displaystyle{{{r_{i}}{D_{ij}}} \over {{\pi _{i}}{{{p}^{\prime}}_{i}}}}$, ${\hat {E}_{i}} = \displaystyle{{{r_{i}}{E_{i}}} \over {{\pi _{i}}{{{p}^{\prime}}_{i}}}}$ and ${\hat {B}_{i}} = {x_{i}} - {\hat { R}_{r}}{{\rm {u}^{\prime}}_{i}}{{\hat { \beta }}_{r}}$.

Next, we consider ${\hat {Y}}_{R}^{\prime (3)}$ under the MCAR mechanism as follows. The new ratio estimator for population mean when $\bar X$ is unknown and nonresponse occurs on both $y$ and $x$ variables under the MCAR mechanism is

(48) $$\hat {\bar Y}_{R}^{\prime\prime(3)} = \displaystyle{{\displaystyle{1 \over N}\sum\limits_{i \in s} {\displaystyle{{{r_{i}}{y_{i}}} \over {{\pi _{i}}p}}} } \over {\displaystyle{1 \over N}\sum\limits_{i \in s} {\displaystyle{{{r_{i}}{x_{i}}} \over {{\pi _{i}}p}}} }}\hat {\bar X}_{GREG}\prime = \displaystyle{{\displaystyle{1 \over N}\sum\limits_{i \in s} {\displaystyle{{{r_{i}}{y_{i}}} \over {{\pi _{i}}}}} } \over {\displaystyle{1 \over N}\sum\limits_{i \in s} {\displaystyle{{{r_{i}}{x_{i}}} \over {{\pi _{i}}}}} }}\hat {\bar X}_{GREG}\prime ,$$

where ${\hat{\bar{X}}}_{GREG}{\prime} = \hat {\bar {X}}_{r}{\prime} + {\left[ {\bar { U} - \hat{\bar{ {U}}}_{r}{\prime}} \right]\prime }{{\hat { \beta }}}_{r}{\prime}$, $\hat {\bar {X}}_{r}{\prime} = \displaystyle{1 \over N}\sum\limits_{i \in s} {\displaystyle{{{r_{i}}{x_{i}}} \over {{\pi _{i}}p}}} ,$ $\hat{\bar { U}}_{r}^{} = \displaystyle{1 \over N}\sum\limits_{i \in s} {\displaystyle{{{r_{i}}{{ u}_{i}}} \over {{\pi _{i}}p}}}$, $\bar { U} = \displaystyle{1 \over N}\sum\limits_{i \in U} {{{ u}_{i}}}$, ${{\hat { \beta }}{\prime}}_{r} = {\left( {\sum\limits_{i \in s} {\displaystyle{{{r_{i}}{q_{i}}{{ u}_{i}}{{{ {u}{\prime}}}_{i}}} \over {{\pi _{i}}p}}} } \right)^{ - 1}}\left( {\sum\limits_{i \in s} {\displaystyle{{{r_{i}}{q_{i}}{{ u}_{i}}{w_{i}}} \over {{\pi _{i}}p}}} } \right)$.

Then, the new ratio estimator for population mean is

(49) $${\hat {Y}}_{R}^{\prime\prime(3)} = N\hat{ \bar Y}_{R}^{\prime\prime(3)}.$$

The variance and associated estimators of ${\hat {Y}}_{R}^{\prime \prime(3)}$ are discussed in Lemma 5.

***Lemma 5*.** *Under condition* $({B_3})$ *with a reverse framework and where the nonresponse mechanism is MCAR*.

(1) The variance of ${\hat {Y}}_{R}^{\prime\prime(3)}$ is

(50) $$V\left( {{\hat {Y}}_{R}^{\prime\prime(3)}} \right) = \sum\limits_{i \in U} {{D_{i}}} B_{i}^2 + \sum\limits_{i \in U} {\sum\limits_{i\backslash \{ j\} \in U} {{D_{ij}}{B_{i}}{B_{j}}} } + \sum\limits_{i \in U} {{E_{i}}B_{i}^2},$$

where ${D_{i}} = \displaystyle{{1 - {\pi _{i}}} \over {{\pi _{i}}}}$, ${D_{ij}} = \displaystyle{{{\pi _{ij}} - {\pi _{i}}{\pi _{j}}} \over {{\pi _{i}}{\pi _{j}}}}$, ${{E}{\prime}_{i}} = \displaystyle{{1 - p} \over p}$ and ${B_{i}} = {x_{i}} -  R{{ {u}{\prime}}_{i}}{ \beta }$.

(2) The estimator of $V\left( {{\hat {Y}}_{R}^{\prime \prime(3)}} \right)$ is

(51) $$\hat {V}\left( {{\hat {Y}}_{R}^{\prime \prime(3)}} \right) = \sum\limits_{i \in s} {{{\hat {D}}_{i}}} \hat {B}_{i}^2 + \sum\limits_{i \in s} {\sum\limits_{i\backslash \{ j\} \in s} {{{\hat {D}}_{ij}}{{\hat {B}}_{i}}{{\hat {B}}_{j}}} } + \sum\limits_{i \in s} {{{{\hat {E}}}_{i}{\prime}}\hat {B}_{i}^2},$$

where ${\hat {D}_{i}} = \displaystyle{{{r_{i}}{D_{i}}} \over {{\pi _{i}}{p}{\prime}}}$, ${\hat {D}_{ij}} = \displaystyle{{{r_{i}}{D_{ij}}} \over {{\pi _{i}}{p}{\prime}}}$, ${\hat {E}_{i}} = \displaystyle{{{r_{i}}{{{E}{\prime}}_{i}}} \over {{\pi _{i}}{p}{\prime}}}$ and ${\hat {B}_{i}} = {x_{i}} - {{\hat { R}}{\prime}_{r}}{{ {u}{\prime}}_{i}}{{\hat { \beta }}{\prime}}_{r}$. The value of ${p}{\prime}$ is defined in (35).

### Simulation studies

In this section, the performance of the proposed new ratio estimators and their variance estimators under the MAR mechanism is compared with the MCAR mechanism *via* simulation studies. We generated a study variable ${y_{i}}$ from the auxiliary variables ${x_{i}}$, ${w_{i}}$, size variable ${k_{i}}$ and calibration variable ${u_{i}}$ following the model from Sichera (2020) and it is defined by ${y_{i}} = 0.2{x_{i}} + 0.1{w_{i}} + 2k + 3.7{k^{\textstyle{1 \over 2}}} + 2{u_{i}} + {\varepsilon _{i}}$ where ${k_{i}}\sim gamma(10,5)$, ${w_{i}}\sim gamma(5,10)$, ${\varepsilon _{i}}\sim N(0,1)$, $\left( \matrix{{x_{i}} \hfill \cr {u_{i}} \hfill} \right)\sim N\left[ {\left( \matrix{15 \hfill \cr 5 \hfill} \right),\left( {\matrix{ 1 & \rho \cr \rho & 1 \cr } } \right)} \right]$, $\rho = 0.70$, $i = 1,2,,...,N$. Four levels of sample sizes $n = 100,200,600$ and 1,200 are drawn from a population size $N = 3,000$ and $n = 10,20,60$ and 1,200 are drawn from a population size $N = 300$ using Midzuno’s (1952) scheme. We consider the MAR response mechanism with two levels of response rate; 60% and 80% and repeated the simulation 10,000 times ( $M = 10,000$) using Program R (R Core Team, 2021). We consider the case where the response probability is unknown and estimated by the logistic regression model for the MAR mechanism and estimated by the function $\hat {p} = \left( {\sum\limits_{i \in s} {\displaystyle{{{r_{i}}} \over {{\pi _{i}}}}} } \right){\left( {\sum\limits_{i \in s} {\displaystyle{1 \over {{\pi _{i}}}}} } \right)^{ - 1}}$ for the MCAR mechanism. The relative root mean square error ( $RRMSE$) was used to compare the efficiency of the proposed ratio estimators and their variance estimators and the formula is

$RRMSE\left( {\hat {V}(\hat {Y})} \right) = \displaystyle{{\sqrt {\displaystyle{1 \over {M - 1}}\sum\limits_{m = 1}^M {{{(\hat {A} - A)}^2}} } } \over A}$

where $\hat {A}$ is the proposed estimators or variance estimators and $A$ is expectation of $\hat {A}$ or $E(\hat {A})$. The results are shown in Tables 1 and 2.

**Table 1:** The relative root mean square error of the new ratio estimators and associated variance estimators for *N* = 3,000.

Response rate (%)	*n*	The relative root mean square error of the proposed estimators				The relative root mean square error of the variance estimators			
		$\bar X$ is known		$\bar X$ is known		$\bar X$ is unknown		$\bar X$ is unknown	
		$PRMSE{\left(\hat {Y}_{R}^{\prime (2)}\right)}$		$PRMSE{\left(\hat {Y}_{R}^{\prime (3)}\right)}$		$PRMSE{\left(\hat {V}\left(\hat {Y}_{R}^{\prime (2)}\right)\right)}$		$PRMSE{\left(\hat {V}\left(\hat {Y}_{R}^{\prime (3)}\right)\right)}$	
		MAR	MCAR	MAR	MCAR	MAR	MCAR	MAR	MCAR
60	100	0.0470	0.0472	0.0465	0.0467	0.1702	0.1703	0.2763	0.3201
	200	0.0350	0.0351	0.0354	0.0361	0.1321	0.1316	0.1761	0.2069
	600	0.0322	0.0324	0.0330	0.0340	0.1150	0.1158	0.1408	0.1658
	1,200	0.0104	0.0105	0.0105	0.0107	0.0427	0.0697	0.0512	0.0804
80	100	0.0364	0.0373	0.0366	0.0375	0.1453	0.1490	0.1930	0.2526
	200	0.0258	0.0261	0.0266	0.0269	0.1127	0.1141	0.1855	0.2048
	600	0.0134	0.0139	0.0146	0.0150	0.0588	0.0614	0.1108	0.1149
	1,200	0.0086	0.0089	0.0088	0.0090	0.0433	0.0552	0.0660	0.0875

**Table 2:** The relative root mean square error of the new ratio estimators and associated variance estimators with population size *N* = 300.

Response rate (%)	*n*	The relative root mean square error of the proposed estimators				The relative root mean square error of the variance estimators			
		$\bar X$ is known		$\bar X$ is unknown		$\bar X$ is known		$\bar X$ is unknown	
		$PRMSE{\left(\hat {Y}_{R}^{\prime (2)}\right)}$		$PRMSE{\left(\hat {Y}_{R}^{\prime (3)}\right)}$		$PRMSE{\left(\hat {V}\left(\hat {Y}_{R}^{\prime (2)}\right)\right)}$		$PRMSE{\left(\hat {V}\left(\hat {Y}_{R}^{\prime (3)}\right)\right)}$	
		MAR	MCAR	MAR	MCAR	MAR	MCAR	MAR	MCAR
60	10	0.1166	0.1196	0.1169	0.1198	0.6605	0.6902	2.2661	2.7669
	20	0.1038	0.1056	0.1049	0.1067	0.5423	0.5452	1.3307	1.3510
	60	0.0624	0.0626	0.0631	0.0634	0.2420	0.2489	0.3666	0.4646
	120	0.0478	0.0479	0.0483	0.0485	0.1548	0.1829	0.2500	0.2634
80	10	0.0949	0.0962	0.0957	0.0968	0.4347	0.4533	2.2106	2.5923
	20	0.0863	0.0869	0.0869	0.0871	0.3722	0.3736	1.3123	1.3319
	60	0.0480	0.0484	0.0481	0.0485	0.2084	0.2150	0.3628	0.4144
	120	0.0298	0.0301	0.0298	0.0303	0.1392	0.1514	0.2164	0.2325

The simulation results found in Table 1 for $N = 3,000$ that the new population total estimator under missing at random performed better than the estimators under missing completely at random for both situations where $\bar X$ is either known or unknown. There was an increase of response rate, decrease of the relative root mean square errors as same as for the sample sizes for all estimators. When $\bar X$ is unknown and needs to be estimated, it results in increasing the relative root mean square errors due to the estimation process. Similar results were found in the case of variance estimators. Similar results are found in Table 2 for a smaller sample size $N = 300.$

### An application to water demand in Thailand

The new estimators are applied to estimate the water demand in Thailand. The data are from the provincial waterworks during August and July 2022. Midzuno’s (1952) scheme is instigated to select a sample of size 40 provinces from the total of 74 provinces. The demand for water in August 2022 is considered as study variable $y$. Two auxiliary variables $x$ and $w$ are the water supply in August and the water demand in July 2022, respectively. The variable $x$ is used to construct the new ratio estimators and the variable $w$ is used to estimate the response probabilities with the logistic regression model under the MAR mechanism. The calibration variable $u$ is the water supply in July 2022 and the size variable $k$ is the number of water users in August 2022. The nonresponse rate is 7.5% in this study.

Table 3 shows the total estimate of water demand in August 2022, Thailand. We see that the estimated water demand when $\bar X$ is known is higher than when $\bar X$ is unknown under both the MAR and MCAR nonresponse mechanisms. In contrast, the estimates of variance when $\bar X$ is unknown is a lot higher than the estimates of variance when $\bar X$ is known due to the estimation of the unknown population mean of the auxiliary variable. The new estimators can be useful for application to the real world when nonresponse occurs in the study which requires management before the estimation process and further analysis.

**Table 3:** The total estimates of water demand in August 2022.

Nonresponse mechanism	Information on the auxiliary variable	Estimated water demand	Variance estimates
MAR	$\bar X$ is known	122,763,533	5,621,837,076,813
	$\bar X$ is unknown	112,079,391	49,945,263,902,570
MCAR	$\bar X$ is known	122,752,240	5,958,276,721,564
	$\bar X$ is unknown	111,926,154	52,711,445,906,451

**Figure 1:**
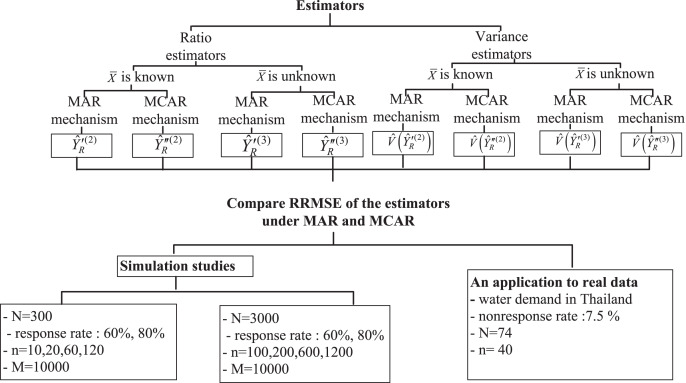
The conclusion for all the cases of the simulation studies in an empirical study.

Figure 1 shows the conclusion for all the cases of the simulation studies in an empirical study.

## Conclusions

The new ratio estimators for estimating population total and population mean when missing data is missing at random occurs with both study and auxiliary variables under UPWOR when the population mean of an auxiliary variable is known and unknown are proposed. In the latter we suggested to estimate it from other variables using the GREG estimator. The new ratio estimators are compared by their efficacies under the MAR and MCAR nonresponse mechanisms through simulation studies and an empirical study using water demand data in Thailand. The results found that the new ratio estimators under the MAR mechanism are more efficient than ratio estimators under the MCAR mechanism for all response rates and sample sizes. The proposed estimators are applied to estimate the demand for water so this information can be used to plan for policies and strategies for preventing water shortages which may occur in the future. The proposed estimators are more useful in practice when compared to the estimators proposed by Ponkaew & Lawson (2018) that considered only under MCAR and when only the study variable is missing which also required the known parameter of the population mean of the auxiliary variable which is difficult to find. The proposed estimators are more flexible to apply in real life because we can use them in more flexible situations when both the nonresponse mechanism is uniform or not uniform which is more likely to occur in real world problems. If the population mean of the auxiliary variable is unknown, it can be estimated using the GREG estimator which makes use of the benefit of the related variables in the estimation process to improve the efficiency of the population total estimators. We can extend the new estimator to complex survey designs such as stratified cluster sampling and consider it under the not missing at random nonresponse mechanism (NMAR).

## Supplemental Information

10.7717/peerj.14551/supp-1 Supplemental Information 1 Code for Simulation Studies.

10.7717/peerj.14551/supp-2 Supplemental Information 2 Code for water demand data.

## Additional Information and Declarations

### Data Availability

The following information was supplied regarding data availability: The data and codes for the simulation studies and an application to water data are available in the Supplemental Files.
